# Involvement of *PtPHR1* in phosphates starvation-induced alkaloid biosynthesis in *Pinellia ternata* (Thunb.) Breit

**DOI:** 10.3389/fpls.2022.914648

**Published:** 2022-08-10

**Authors:** Huihui Wang, Jitao Hu, Linying Li, Xueying Zhang, Hao Zhang, Zongsuo Liang, Qing Sheng, Yuqing He, Gaojie Hong

**Affiliations:** ^1^College of Life Sciences, Zhejiang Sci-Tech University, Hangzhou, China; ^2^State Key Laboratory for Managing Biotic and Chemical Threats to the Quality and Safety of Agro-Products, Institute of Virology and Biotechnology, Zhejiang Academy of Agricultural Sciences, Hangzhou, China; ^3^College of Pharmacy, Zhejiang Chinese Medical University, Hangzhou, China

**Keywords:** *Pinellia ternata* (Thunb.) Berit, alkaloid metabolism, benzoic acid (BA), phosphate signaling, *PtPHR1*

## Abstract

Nowadays, because of the great benefit to human health, more and more efforts have been made to increase the production of alkaloids in *Pinellia ternata* (Thunb.) Breit. Phosphate (Pi) plays a critical role in plant growth and development, as well as secondary metabolism. However, its effect and regulation mechanism of Pi signaling on alkaloid biosynthesis call for further exploration. Here, we reported that Pi starvation could induce alkaloid accumulation in *P. ternata*. We cloned a cDNA sequence encoding PtPHR1 from *P. ternata*, which was further identified by nuclear localization, transcription activity, and binding ability to the PHR1-binding sequence. We found that the transformation of *PtPHR1* into the *Arabidopsis phr1* mutant (designated as *PtPHR1OE/phr1*) led to the rescue of the phenotype of the *phr1* mutant to that of the wild-type, including the expression level of Pi starvation-induced genes and anthocyanin accumulation. The combination of these biochemical and genetic experiments indicated that PtPHR1 was intended to have a role similar to that of AtPHR1 in Pi signaling and metabolic responses. Interestingly, we found that Pi starvation also induced the production of benzoic acid, an intermediate in the biosynthetic pathway of phenylpropylamino alkaloids. Furthermore, this induction effect was impaired in the *phr1* mutant but partly recovered in *PtPHR1OE/phr1* plants. Together, our data suggest that Pi starvation promoted benzoic acid-derived alkaloid biosynthesis in *P. ternata* under the control of PtPHR1. Our finding that *PtPHR1* is involved in the regulation of Pi signaling on alkaloid biosynthesis shows a direct link between the Pi nutrient supply and secondary metabolism.

## Introduction

*Pinellia ternata* (Thunb.) Breit, a member of the *Araceae* family, is an essential Chinese traditional herb with a long history of use for medical treatment (Mao and He, 2020). The dried tuber of this herb, called “banxia” in Chinese, is the major part of the plant used for medicine. *P. ternata* has broad pharmacological and clinical properties such as, wound healing, cough soothing, anti-spasmodic, anti-tumoral, and lipid-lowering effects (Wang et al., 2009; Wu et al., 2013; Ji et al., 2014; Mao and He, 2020). *P. ternata* is abundant in secondary metabolites (i.e., alkaloids, iridoids, iridoid glycosides, anthraquinones, anthraquinone glycosides, sterols, amino acids, and fatty acids), as well as their derivatives (Oshio et al., 1978; Ge and Hao, 2009; Sun et al., 2018). Among them, alkaloids are the main biologically active compounds with anti-tumoral and anti-viral activities (Xu et al., 2007; Ji et al., 2014). According to the Chinese Pharmacopoeia Commission (2005), ephedrine and purine, two main active ingredients of alkaloids, are quality markers of *P. ternata*. It is apparent that the supply of alkaloids in *P. ternata* cannot keep up with the growing demand for them any longer (Liu et al., 2015; Xue et al., 2019; Mao and He, 2020). Therefore, a deeper and more comprehensive understanding of the alkaloid synthesis pathway and its regulatory mechanism would aid in improving the supply.

To date, two potential pathways of phenylpropylamino alkaloids biosynthesis have been characterized and identified, namely, the β-oxidative and non-β-oxidative routes (Boatright et al., 2004; Zhang et al., 2016; Duan et al., 2019). Respectively, the main enzymes involved in the β-oxidative pathway are phenylalanine ammonia lyase (PAL), cinnamate: CoA ligase (CNL), cinnamoyl CoA hydratase-dehydrogenase (CHD), and 3-ketoacyl-CoA thiolase (KAT) (Klempien et al., 2012). In the non-β-oxidative pathway, sequential catalyzation by 3-hydroxyisobutyryl-CoA hydrolase (CHY), benzaldehyde dehydrogenase (BALDH) and aldehyde oxidase 4 (AO4) (Van et al., 2009) was responsible for the formation of benzoic acid (BA). Followed by ThDP-dependent pyruvate decarboxylase (ThPDC) and acetolactate synthase (AHAS), the BA was further converted into 1-phenylpropane-1,2-dione (Müller et al., 2009). Using RNA-seq data, a comprehensive genome of *P. ternata* has been provided (Zhang et al., 2016; Tian et al., 2022). However, the genes and signaling pathways involved in the regulation of alkaloid biosynthesis need to be further elucidated.

The essential element phosphorus is indispensable in and critical for plant growth and development (Cong et al., 2020). In soil, the concentration of soluble inorganic phosphate (Pi) is relatively low, and as a result, Pi starvation seriously impacts and limits plant growth and yield (Neumann and Römheld, 2012). To adapt to Pi starvation, plants change the architecture of their root systems so they have a shorter primary root and higher root density, enhance the root's exudation, and increase the expression of Pi transporter genes and they also use other strategies to facilitate Pi acquisition (Yuan and Dong, 2008). In recent decades, the Pi signaling pathway has been well elucidated and most components involved have been identified (Yuan and Dong, 2008; Crombez et al., 2019). Among them, phosphate starvation response proteins (PHRs) serve as the key regulators of the Pi starvation signal. As reported, PHRs can recognize a specific DNA motif (P1BS, sequence GNATATNC) and regulate most Pi starvation-induced (PSI) genes (Rubio et al., 2001). The SPX protein family, made up of a single SYG1/Pho81/XPR1 (SPX) domain, suppresses Pi starvation signaling by interacting with PHRs and inhibiting their transcriptional activities (Lv et al., 2014; Ruan et al., 2019).

Until now, several studies have implicated Pi starvation signaling in the biosynthesis of secondary metabolites. In *Arabidopsis*, the *phr1* mutant ablates Pi starvation-induced anthocyanin production whereas the *spx1spx2* double mutant accumulates more anthocyanins in response to Pi starvation (Rubio et al., 2001; Puga et al., 2014). Most recently, we reported that SPX4 could interact with both PHR1 and PAP1 to regulate Pi starvation-mediated flavonoid biosynthesis (He et al., 2021). Pi deficiency also affects the accumulation of indole glucosinolates in *Arabidopsis* co-cultivated with *Colletotrichum tofieldiae* (Frerigmann et al., 2021). In addition, the Pi supply could affect chicoric acid accumulation in the hairy roots of *Echinacea purpurea* (Salmanzadeh et al., 2020). However, the involvement of Pi signaling in the regulation of alkaloid biosynthesis and its molecular mechanism has yet to be reported.

Herein, we demonstrated that Pi starvation increased the transcript level of alkaloid biosynthetic genes and thereby promoted alkaloid accumulation. Based on the cloning and characterizing of the *PtPHR1* sequence of *P. ternata*, we identified its nuclear localization, transcriptional activity, and recognition of the P1BS element. Through heterologous expression of *PtPHR1* in the *Arabidopsis phr1* mutant, we further demonstrated that PtPHR1 regulated Pi-starvation induced BA biosynthesis. Together, our results revealed that Pi signaling mediated alkaloid biosynthesis, which was under the control of PtPHR1.

## Materials and methods

### Plant materials

**Figure 1:**
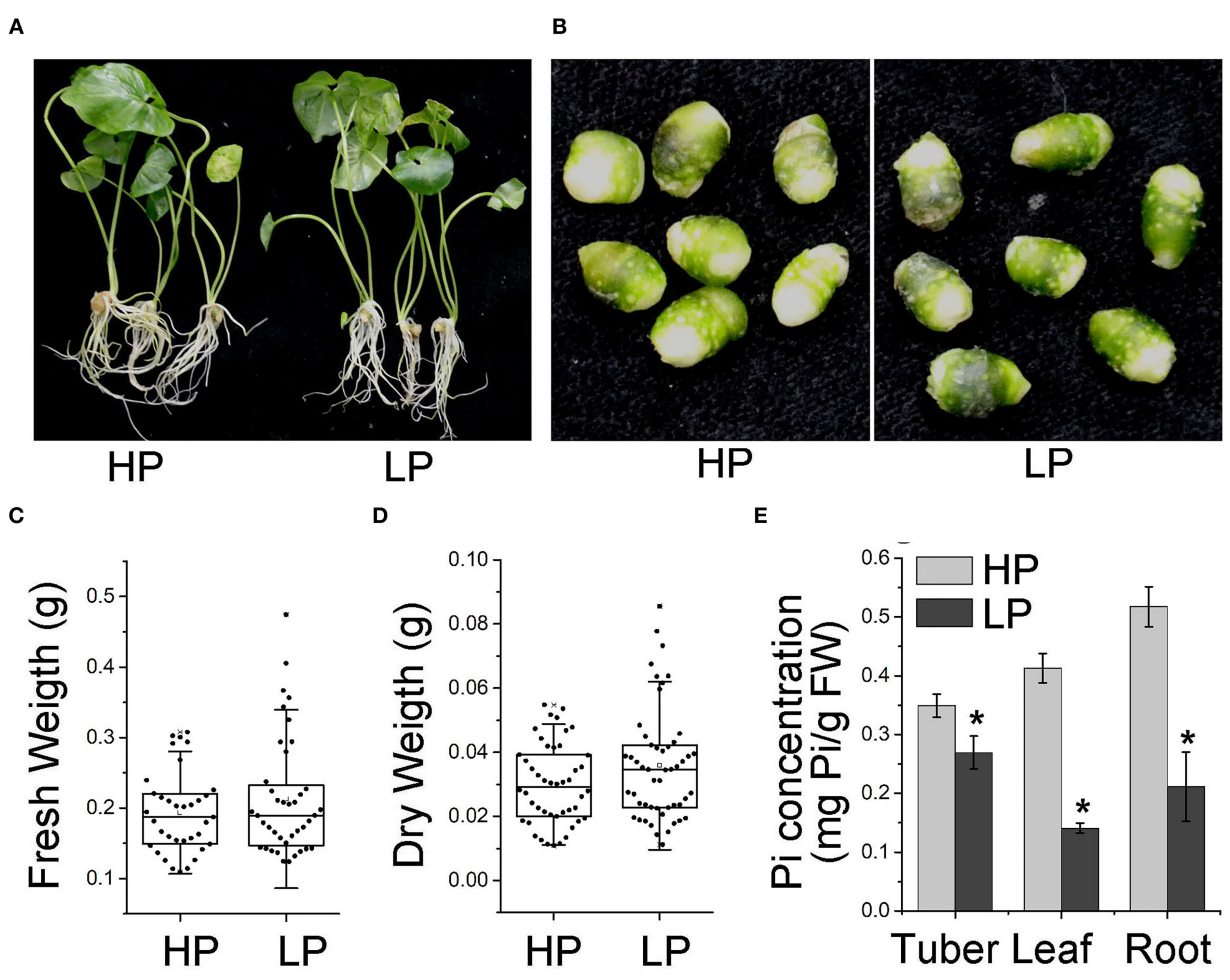
Phenotype of *P. ternata* in conditions of high and Low Pi. Phenotype of *P. ternata* whole plants **(A)** and tubers **(B)** cultured in HP and LP medium for 7 days. **(C,D)** Effect of Pi starvation on the tubers of *P. ternata* in terms of wet **(C)** and dry **(D)** weight. **(E)** Pi concentration in the tubers, leaves, and roots of *P. ternata* upon Pi starvation treatment for 7 days. Values are means ± SD of three biological replicates. *P < 0.05, Student's *t*-test. HP: high Pi, 10 mM. LP: low Pi, 10 μM.

Tubers of *P. ternata* were washed, sliced, and cultured at 25°C with a 16 h/8 h-light/dark photoperiod as described (Liu et al., 2010). For Pi starvation, 30-day-old *P. ternata* (Figure 1A) was cultured in half-strength liquid MS (Murashige and Skoog) medium. The medium was prepared with MS without phosphate (MSP11-50LT; Caisson Laboratories, USA), 0.05% (w/v) MES, and 0.6% (w/v) sucrose. In addition, the high Pi medium was supplemented by 10 mM KH_2_PO_4_, 10 μM KH_2_PO_4_, and equimolar amounts of KCl (Khan et al., 2016; Kong et al., 2021) were added to the low Pi medium. During treatment, the nutrient solution was replaced every 3 days.

Surface-sterilized *Arabidopsis thaliana* ecotype Columbia-0 (Col-0) and *phr1* mutants were germinated on half-strength MS and grown in a growth chamber at 22°C with a 16 h/8 h-light/dark cycle. To generate the *PtPHR1-myc* constructor, the full-length ORF of *PtPHR1* was cloned and inserted into a modified *pCAMBIA1300* vector with an N-terminal fusion 6xmyc tag under the control of the 35S promoter (He et al., 2021). The *PtPHR1-myc* was then introduced into *phr1* mutants by the *Agrobacterium*-mediated flower-dip method to generate *PtPHR1OE/phr1* plants. Hygro-F/R (hygromycin B phosphotransferase detection) and *PtPHR1-detect-F/R* were used to identify homozygous lines, and anti-myc antibody was used to measure the PtPHR1-myc protein level. The primers used in this study are listed in Supplementary Table S1.

### Determination of Pi content

The tubers, leaves, and roots of *P. ternata* grown in a high and low Pi medium for 7 days were collected respectively for Pi measurement, according to the procedure previously described (Zhou et al., 2008).

### Total RNA extraction and RT-qPCR

A total of 1 μg of RNA was used to synthesize the first-strand cDNA, using the HiScript II Q RT for qPCR (+gDNA viper) kit (Vazyme, China). Then RT-qPCR (Hieff qPCR SYBR Green Master Mix, Yeasen, China) was performed on a QuantStudio™ 6 Flex Real-Time PCR System (Applied Biosystems, Singapore), following the manufacturer's instructions. *Pt18S* was used as a reference for normalization, and expression levels were analyzed by the comparative Ct method (2^−ΔΔCt^ method). At least three biological replicate samples were included. The primers used in this study are listed in Supplementary Table S1.

### Determination of alkaloid and flavonoid content

Standard chemicals, including ephedrine hydrochloride (171241-201809), guanosine (111977-201501), adenosine (110879-201703), and uridine (110887-202104) were purchased from the National Institute for the Control of Pharmaceutical and Biological Products, Beijing, China, and BA (PHR1050), validated by 1H Nuclear Magnetic Resonance (NMR, D_2_O) spectroscopy on Bruker AVANCE III 500 MHz with TMS, were purchased from Sigma–Aldrich, Shanghai, China.

Tubers of *P. ternata* grown under high and low Pi conditions were completely dried at 60°C until they reached a constant weight. The dried tubers were ground into powder and then used for alkaloid extraction. The total alkaloid content was determined by using a UV–visible spectrophotometer at a wavelength of 416 nm according to a previous protocol (Liu et al., 2010; Duan et al., 2019). Guanosine, adenosine, and uridine were determined at 240 nm (Ji et al., 2013), and BA at 230 nm (Iwakoshi et al., 2019). High-performance liquid chromatography (HPLC) analysis was performed using a Shimadzu Prominence-I LC-2030C 3D Plus with a 5 μm C18 column (five-particles, 4.6 × 150 mm).

The total flavonoids were extracted and measured by the Flavonoid Extraction Kit (LHT-1-G, Cominbio, China). The anthocyanin content was extracted and determined at the valve of A_657_-A_530_.

### Isolation of *PtPHR1* and sequence analysis

The *PtPHR1* ORF sequence was amplified using the rapid amplification of the cDNA ends method. The conserved domains were analyzed using the NCBI database (www.ncbi.nlm.nih.gov/Structure/cdd/wrpsb.cgi). Multiple alignments were analyzed using ClustalW (http://clustalw.ddbj.nig.ac.jp/). Phylogenetic and molecular evolutionary analysis was conducted using MEGA 6.0 software.

### Subcellular localization analysis

The *PtPHR1* ORF was cloned into the *pCV-GFP-N1* binary vector to generate the *pCV:PtPHR1-GFP* construct. The *pCV:PtPHR1-GFP* was transiently expressed in *Nicotiana benthamiana* leaves by *Agrobacterium* tumefaciens infiltration (He et al., 2020). Using a TCS SP5 confocal laser scanning microscope system, the fluorescence signal was detected (Leica Microsystems, Bannockburn, IL, USA) at 40–44 h after infiltration.

### Transcriptional activation

The transcriptional activation assay was conducted as described (Qiao et al., 2017). The ORFs of *PtPHR1* and *AtPHR1* were cloned and inserted into the vector pGBKT7 (BD) to obtain *BD-PtPHR1* and *BD-AtPHR1*, respectively. The BD-AtPHR1 was used as a positive control and the empty BD vector as a negative control. All these vectors were transformed into AH109, grown on –Trp medium, and then selected on the –Trp/–His medium with 3-amino-1,2,4-triazole (3-AT). The primers used are listed in Supplementary Table S1.

### Y1H assay

For the Y1H assay, the 4×P1BS sequence was inserted into the pHis2.1 vector. The ORFs of *PtPHR1* and *AtPHR1* were cloned and co-transformed with the SmaI-linearized pGADT7-Rec2 vector into the yeast strain Y1HGold, respectively. Th colonies were incubated at 30°C on SD medium lacking Leu and Trp, and then spotted on –His/–Leu/–Trp minimal medium with 0, 5, 50 mM 3-AT, respectively. The primers used are provided in Supplementary Table S1.

### Statistical analysis

For analyzing differences between two variables, the Student's *t*-test was used. When variables were over two, ANOVA with Fisher's least significant difference test was adopted. A *p*-value < 0.05 was considered statistically significant. All analysis was performed using ORIGIN 8 software.

## Results

### Extracellular phosphate limiting resulted in intracellular Pi deficiency

To investigate the effect of Pi starvation on *P. ternata*, we cultured *P. ternata* in liquid media containing low Pi (10 μM, HP) and high Pi (10 mM, LP) (Khan et al., 2016), respectively. After 7 days, there was no sign of toxicity phenotypes at the high Pi level or Pi deficiency phenotypes at the low Pi level (Figures 1A,B). No significant difference was observed in the fresh and dry tuber weight between plants grown in Pi-sufficient and Pi-deficient conditions (Figures 1C,D). However, the Pi concentrations in the tubers, leaves, and roots of *P. ternata* grown in low Pi media were obviously lower than those grown in a high-Pi medium (Figure 1E). These results suggested that the growth of *P. ternata* was not altered during a short period of Pi deficiency.

### The induction of the alkaloid biosynthetic pathway by Pi starvation

**Figure 2:**
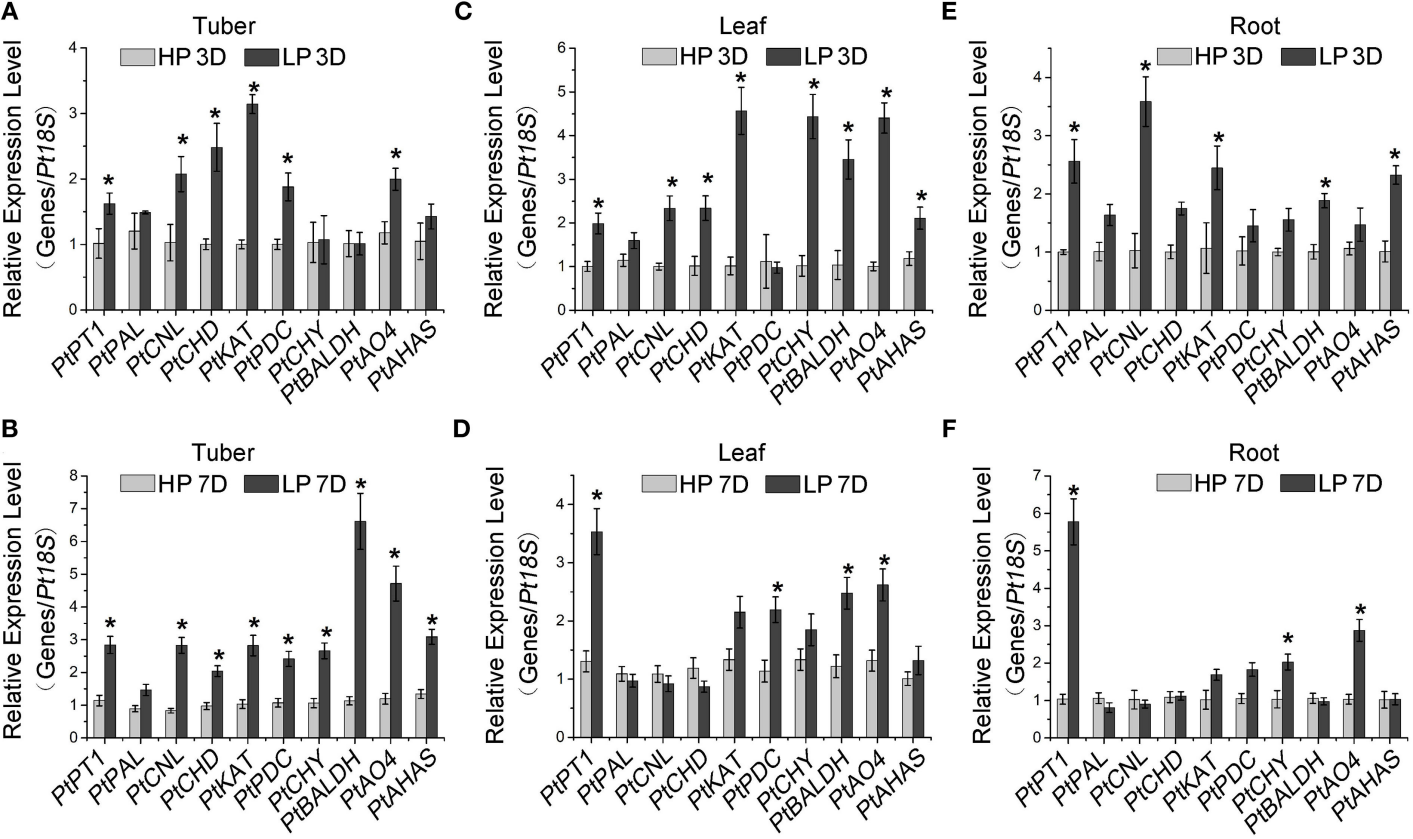
Induction of alkaloid-responsive gene expression by Pi starvation. RT-qPCR analysis of alkaloid-responsive genes in *P. ternata* tubers **(A,B)**, leaves **(C,D)**, and roots **(E,F)** upon low Pi treatment for 3 days **(A,C,E)** and 7 days **(B,D,F)**. *PtPT1* is a marker gene for Pi starvation. Values are means ± SD of three biological replicates. *Indicates a significant difference between high/low Pi treatment at *P* < 0.05 by Student's *t*-test. HP: high Pi, 10 mM. LP: low Pi, 10 μM.

It has been well known that Pi starvation enhances the expression of flavonoid biosynthesis genes and thereby promotes flavonoid accumulation (Rubio et al., 2001; Puga et al., 2014; He et al., 2021). Consistently, we found that the gene expression involved in flavonoid biosynthesis, such as that of *PtCHS, PtCHI*, and *PtF3H* (Zhang et al., 2016; Xue et al., 2019), significantly increased in Pi-starved *P. ternata* compared with the high-Pi control (Supplementary Figures S1A–C), which resulted in a higher total flavonoid accumulation (Supplementary Figure S1D). To explore the effects of Pi on alkaloid biosynthesis, we analyzed the expression of alkaloid biosynthetic genes using RT-qPCR. In *P. ternata* tubers, the expression levels of *PtCNL, PtCHD, PtKAT, PtPDC, PtAO4*, and *PtAHA*S (Zhang et al., 2016) were significantly induced by 3- and 7-day Pi starvation (Figures 2A,B, Supplementary Figure S2). The up-regulation of *PtCHY* and *PtBALDH* was only observed in plants during the 7-day treatment, not during the 3-day (Figures 2A,B). The consistent induction pattern of Pi starvation on alkaloid biosynthetic genes in the leaves and roots of *P. ternata* was also observed (Figures 2C–F).

**Figure 3:**
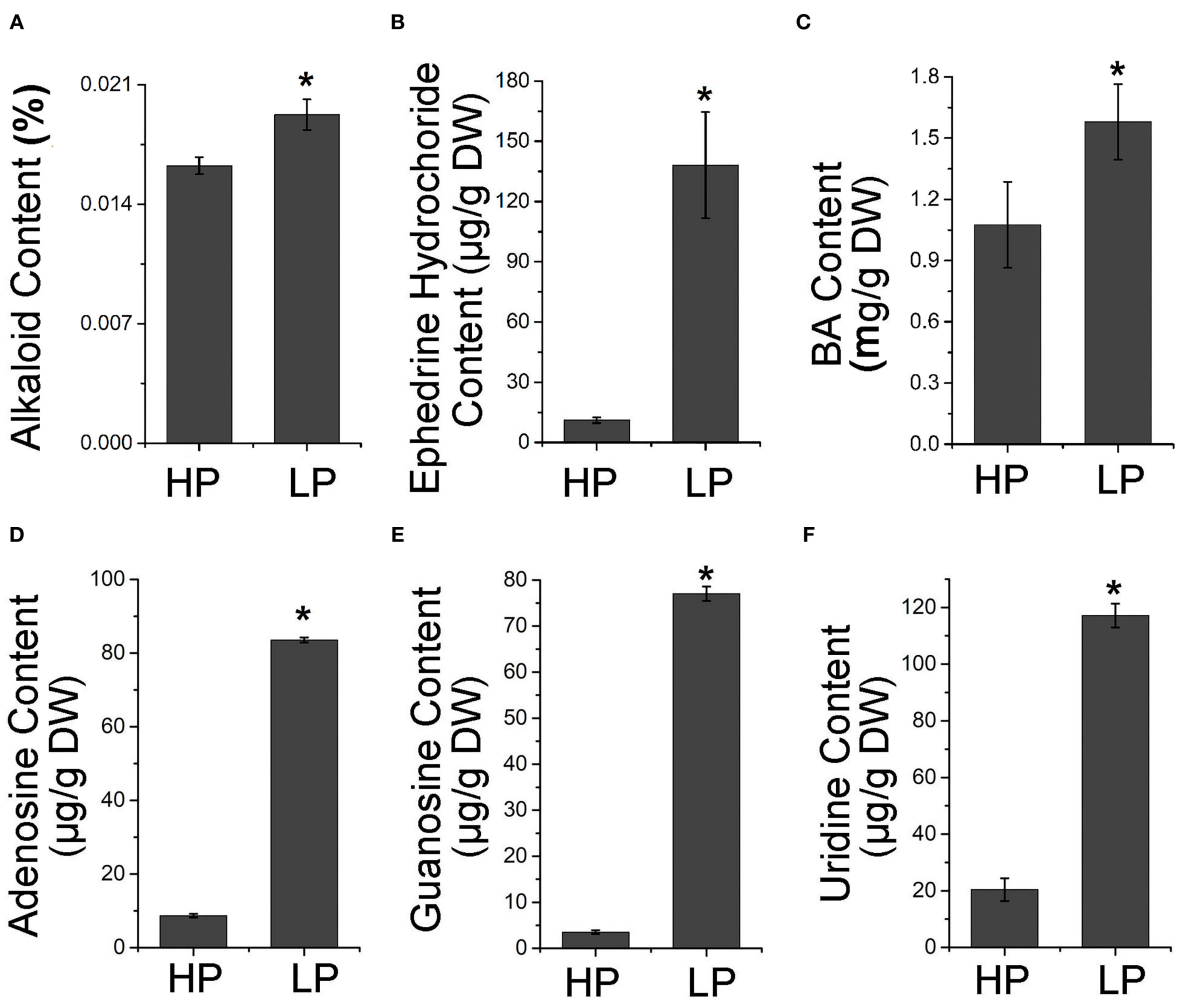
Accumulation of alkaloid compounds by Pi starvation treatment. Contents of total alkaloid **(A)**, ephedrine hydrochloride **(B)**, BA **(C)**, guanosine **(D)**, adenosine **(E)**, and uridine **(F)** in tubers of *P. ternata* grown in high and low Pi for 7 days. Values are means ± SD of three biological replicates. *Indicates a significant difference between high/low Pi treatment at *P* < 0.05 by Student's *t*-test. DW, dry weight. HP: high Pi, 10 mM. LP: low Pi, 10 μM.

To confirm whether the alteration of alkaloid biosynthetic gene expression led to the change in alkaloid production, the alkaloid content in the tubers of Pi-starved plants was measured. The results showed that the total alkaloid content was higher in tubers from plants grown in low Pi conditions than in those from high Pi conditions (Figure 3A). In addition, the content of ephedrine hydrochloride and BA, as well as three purine alkaloids (including adenosine, guanosine, and uridine) was significantly increased to different degrees in Pi-starved conditions (Figures 3B–F, Supplementary Figures S3, S4). These results together suggest that Pi starvation activates the alkaloid pathway.

### Cloning and characterization of *PtPHR1* gene

**Figure 4:**
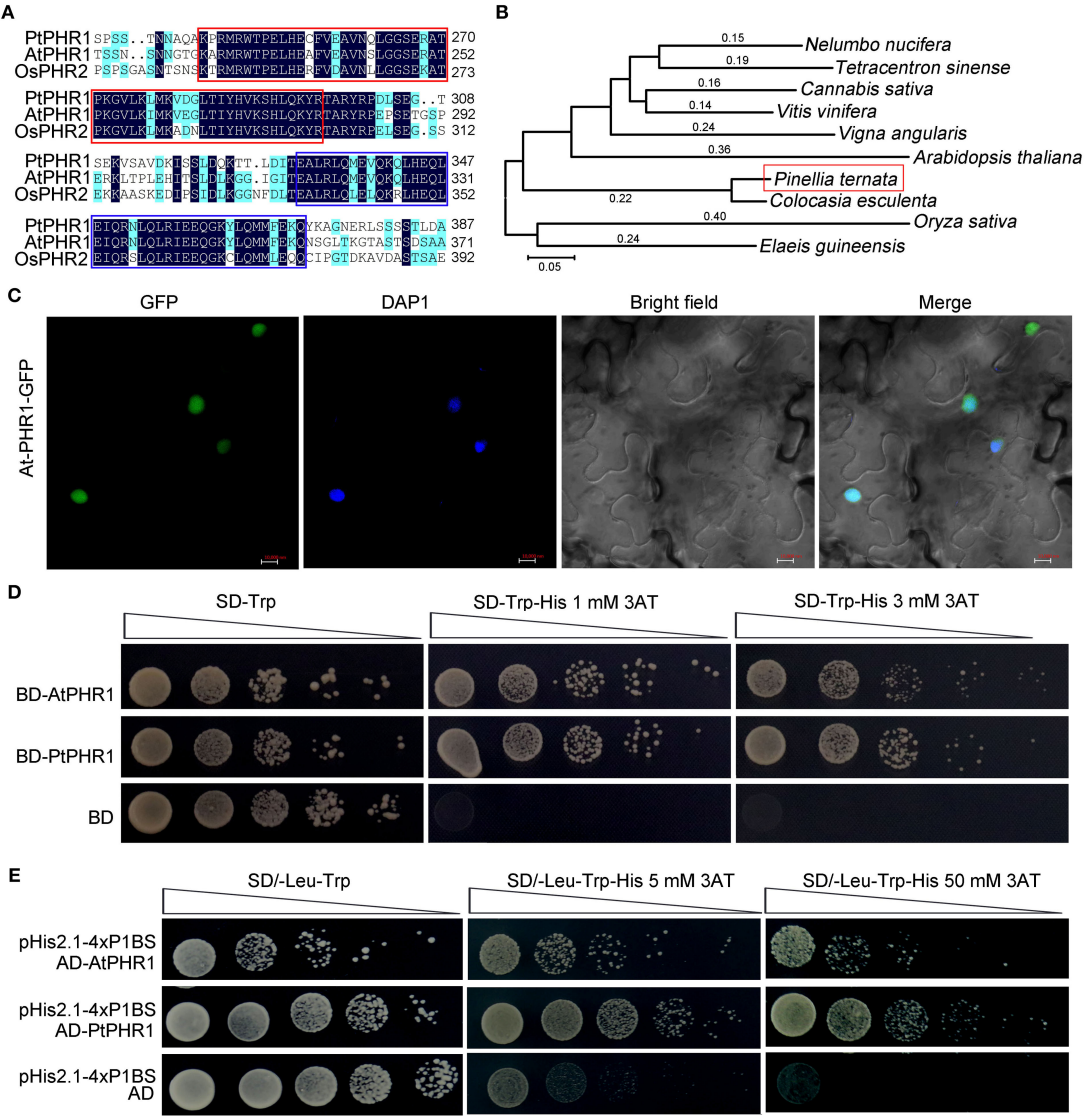
Characterization of PtPHR1 structure, evolution, localization, and activity. **(A)** Multiple alignments of PtPHR1, AtPHR1, and OsPHR2. Similar residues are shaded blue and identical residues are shaded black. The conserved MYB-domain (red) and predicted CC domain (purple) are shown. **(B)** Phylogenetic analysis of PtPHR1 amino acid sequences used the neighbor-joining method. The accession numbers of sequences used are as follows: *Arabidopsis thaliana*, NP_194590.2; *Oryza sativa*, XP_015647735.1; *Nelumbo nucifera*, XP_010264912.1; *Tetracentron sinense*, KAF8394238.1; *Cannabis sativa*, XP_030498437; *Vitis vinifera*, XP_002270511.1; *Vigna angularis*, XP_017411143.1; *Colocasia esculenta*, MQM19898.1 and *Elaeis guineensis*, XP_010920302.1. **(C)** Nuclear localization of PtPHR1-GFP. Bars = 10 μm. **(D)** PtPHR1 has transcriptional activation activity in yeast. The ORF of PtPHR1 was cloned into the vector pGBKT7 (BD). AtPHR1 was used as a positive control. Transformed yeasts were serially diluted and selected on media lacking Trp and His containing 3-aminotriazole (3AT). **(E)** Yeast one-hybrid assays showing interaction between PtPHR2 and P1BS *cis*-element. AtPHR1 was used as a positive control and empty pGADT7-Rec2 (pAD) vectors were used as a negative control.

We cloned and obtained the full-length cDNA of *PtPHR1* (GenBank no. ON075805, https://www.ncbi.nlm.nih.gov/), encoding a predicted protein of 477 amino acids (Supplementary Figure S5). Multiple alignments with AtPHR1 (Rubio et al., 2001) and OsPHR2 (Zhou et al., 2008) revealed that a conserved MYB DNA-binding domain existed in the N-terminal of PtPHR1, and that a coiled–coil (CC) domain was present in its C-terminal (Figure 4A). Phylogenetic tree analysis revealed that PHR1 proteins from *P. ternata* and *Colocasia esculenta* were grouped together, both of which belonged to an *Asteraceae* cluster (Figure 4B). To characterize its features as a transcription factor, we carried out a subcellular localization analysis, showing that the PtPHR1-GFP fusion protein was localized in the nucleus of *N. benthamiana* epidermal cells (Figure 4C). Then, the PtPHR1 was inserted into pGBKT7 (BD) and the BD-PtPHR1 constructor was transformed into the yeast strain AH109 (Qiao et al., 2017). The results showed that PtPHR1 possessed transcription activity, as did the positive control AtPHR1 (Figure 4D). To examine the binding ability of PtPHR1 to the P1BS *cis*-element, we co-transformed pH is 2.1–4× P1BS (bait) and *pGADT7-Rec2-PtPHR1* (prey) into AH109 yeast cells. When spotted on selective media lacking Leu, the colonies grew well with an addition of 3-AT, irrespective of 5 or 50 mM. These results demonstrated that PtPHR1, behaving like AtPHR1, could bind to the P1BS *cis*-element in yeast (Figure 4E). Furthermore, we measured the expression of *PtPHR1* after Pi deficiency and found that the transcript level of *PtPHR1* in the tuber, roots, and leaves was not changed between high and low Pi conditions for 3- or 7-day culture (Supplementary Figure S6).

### PtPHR1 regulates Pi starvation response in *Arabidopsis*

**Figure 5:**
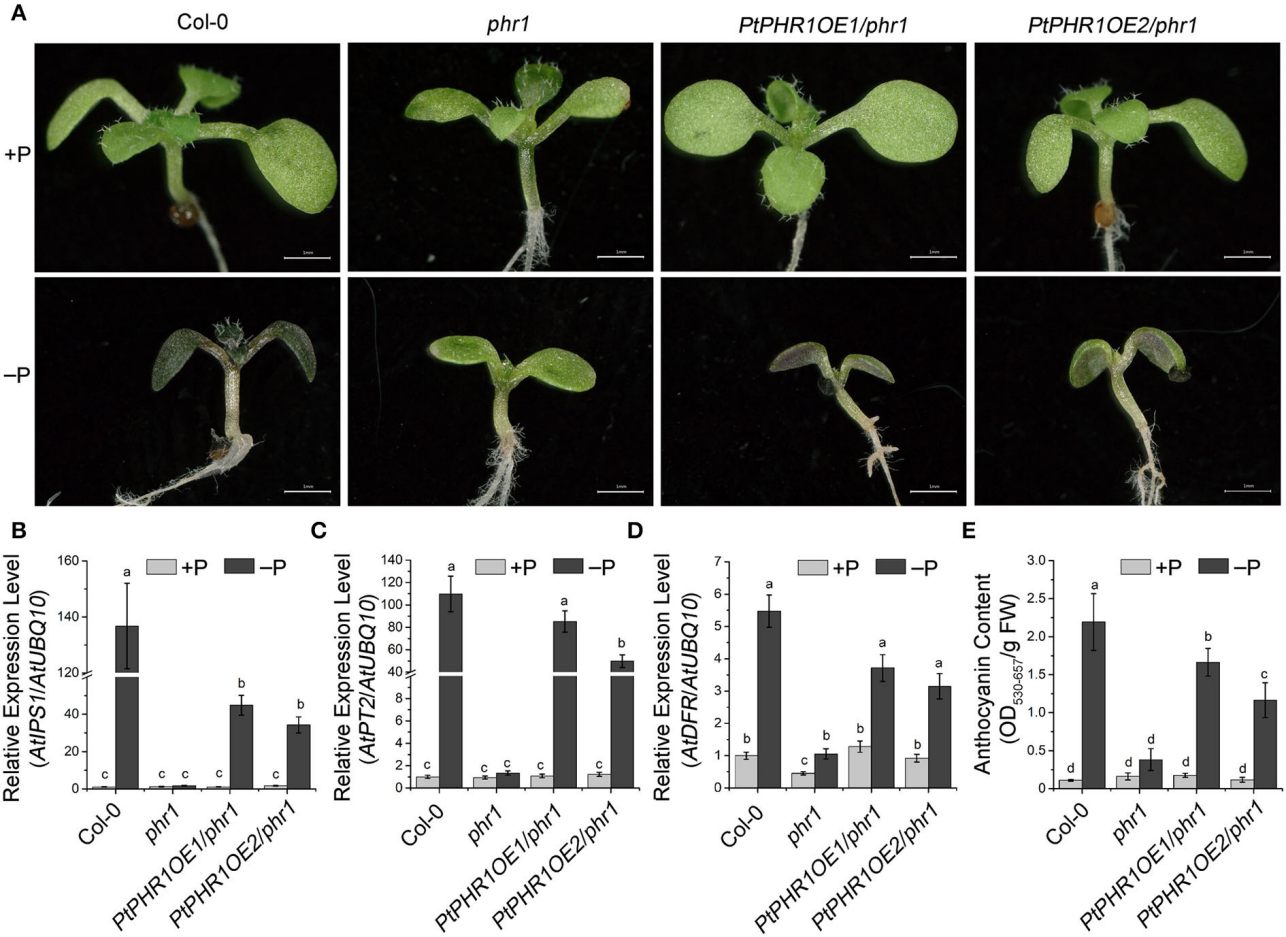
PtPHR1 regulates PSI gene induction and anthocyanin accumulation. **(A)** Images of the 9-day-old Col-0, *phr1*, and *PtPHR1OE/phr1* seedlings grown in +P and –P media. A high-level accumulation of anthocyanins is indicated by the purple color. Bars, 500 mm. **(B–D)** Pi signaling gene and *AtDFR* expression induction levels in the Col-0, *phr1*, and *phr1PtPHR1* seedlings grown in the –P conditions relative to those in +P conditions for 9 days; the transcript levels were analyzed by qRT-PCR. Values are means ± SD of three biological replicates. Different letters indicate significant differences at *P* < 0.05 by Fisher's least significant difference tests. **(E)** Anthocyanin content in the Col-0, phr1, and *phr1PtPHR1* plants are grown in the +P/–P condition for 9 days. Values are means ± SD of three biological replicates. *P* < 0.05, Fisher's least significant difference test.

To further investigate the role of *PtPHR1* in Pi signaling, we transformed PtPHR1 fused with myc-tag into the *phr1* mutant to generate *PtPHR1OE/phr1* transgenic plants (Figure 5A, Supplementary Figure S7). We observed that the phenotype of the *phr1* mutants was partly rescued with the transformation of the PtPHR1-myc plasmid when grown in Pi starvation conditions (Figure 5A). We then analyzed the transcript level of the PSI genes and anthocyanin accumulation in *phr1* and *PtPHR1/phr1* plants grown on low P media. The results showed that the expression of *AtIPS1* and *AtPT2* were elevated by Pi starvation (Figures 5B,C). Besides, *AtDFR* expression in the *PtPHR1OE/phr1* plants was more responsive to Pi starvation compared to that in the *phr1* mutant (Figure 5D). Consistently, the Pi starvation-induced anthocyanin accumulation was recovered in *PtPHR1OE/phr1* plants (Figure 5E). These results indicated that heterologous expression of *PtPHR1* could modulate Pi signaling and anthocyanin biosynthesis in *Arabidopsis*.

### Involvement of PtPHR1 in Pi starvation-induced BA accumulation in *Arabidopsis*

**Figure 6:**
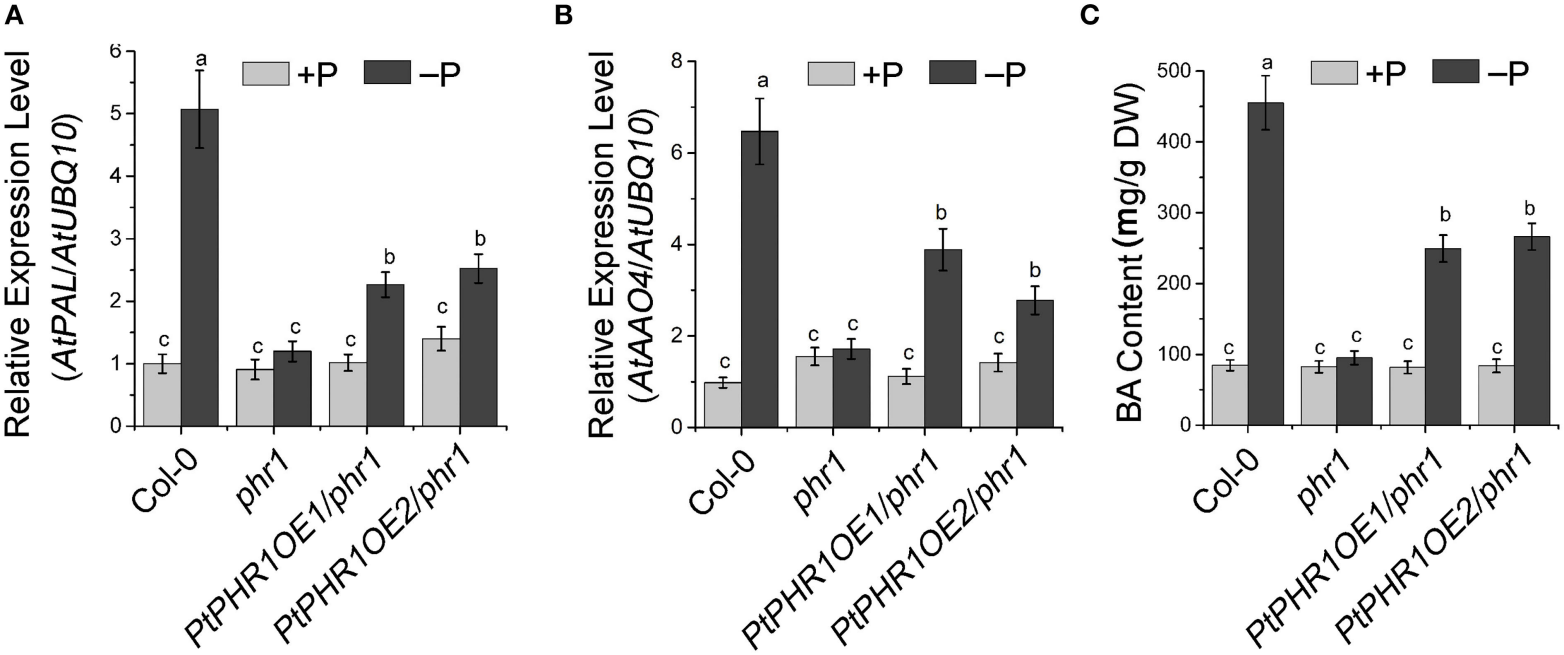
*PHR1* is involved in Pi starvation-induced BA biosynthesis. Expression level of *AtPAL* **(A)** and *AtAAO4* **(B)** and BA content **(C)** in 9-day-old Col-0, *phr1*, and *PtPHR1OE/phr1* seedlings grown in +P and –P conditions. The transcript levels were analyzed by qRT-PCR. Values are means ± SD of three biological replicates. Different letters indicate significant difference at *P* < 0.05 by Fisher's least significant difference test.

In *Arabidopsis*, the homologs of *AtPAL, AtCHY1*, and ARABIDOPSIS ALDEHYDE OXIDASE4 (*AtAAO4*) are involved in BA biosynthesis (Facchini et al., 2004; Ibdah and Pichersky, 2009; Ibdah et al., 2009; Krizevski et al., 2010; Fraser and Chapple, 2011). Given that Pi starvation enhanced BA accumulation in *P. ternata* (Figure 3B), we investigated the impact of Pi signaling and PtPHR1 on alkaloid biosynthesis. Among Col-0, *phr1*, and *PtPHR1OE/phr1* plants, the transcript level of *AtPAL* and *AtAAO4* was strongly increased in Col-0 and *PtPHR1OE/phr1* seedlings grown in Pi-starved medium for 9 days compared with those grown in Pi-sufficient medium, and no obvious induction was observed in *phr1* mutant seedlings (Figure 6A). However, the expression pattern of *AtCHY1* exhibited relatively constant among different genotypes, irrespective of Pi supply (Supplementary Figure S8). Consistently, BA accumulation was enhanced in Col-0 and *PtPHR1OE/phr1* plants but not in the *phr1* mutant (Figure 6B, Supplementary Figure S9). These results demonstrated that PtPHR1, at least partly, regulated Pi signaling-mediated BA biosynthesis.

## Discussion

It has been widely reported that in plants, the regulation of metabolism by Pi signaling is involved in the adaptation to environmental stress (Baek et al., 2017). Pi starvation promotes sugar accumulation, and in turn sucrose elevates the transcript levels of Pi transporters and PSI genes (Hammond and White, 2008). Pi starvation also affects plant hormone biosynthesis, through the effects of auxin (Pérez-Torres et al., 2008), cytokinin (López-Bucio et al., 2002), and gibberellins (Jiang et al., 2007) in modifying the root system architecture formation; and through the effects of jasmonate acid in preventing attacks from insect herbivory and pathogen (Khan et al., 2016; Kong et al., 2021). In addition, Pi starvation results in increased flavonoid accumulation, especially excessive anthocyanin (Bustos et al., 2010; Khan et al., 2016; He et al., 2021). Here, we also observed that Pi-starvation enhanced the expression of flavonoid biosynthesis genes and the accumulation of flavonoids in *P. ternata* (Supplementary Figure S1). Importantly, we demonstrated that Pi starvation could promote alkaloid metabolism in *P. ternata* (Figures 2, 3).

In this work, we characterized the full-length sequence of the *PtPHR1* gene. There were several common features shared by PtPHR1 and other PHR homologs (Rubio et al., 2001; Zhou et al., 2008). Along with the existence of both the MYB domain and CC domain, PtPHR1 was further suggested as a member of the MYB-CC family by sequence comparison analysis (Figure 4A). Based on the chloroplast genome sequence analysis of *P. ternata* and *C. esculenta, PtPHR*1 was identified as a distinct member of the *Asteraceae via* phylogenetic tree analysis (Figure 4B) (Han et al., 2016). In addition, we demonstrated that PtPHR1 was localized to the nucleus and possessed transcription activity (Figures 4C,D), and we pointed out that PtPHR1 could directly bind to the P1BS *cis*-element in yeast (Figure 4E). Furthermore, the steady expression of *PtPHR1* was not very responsive to Pi deprivation (Supplementary Figure S6), consistent with previously reported findings (Rubio et al., 2001; Zhou et al., 2008). Together, these data indicated that PtPHR1 was conserved as plant PHRs and might play an important role in *P. ternata* during the Pi starvation response.

In *Arabidopsis, AtPHR1* has been identified as the core mediator in Pi signaling and as a regulator of PSI genes (Bustos et al., 2010). It has been reported that the mutation of *AtPHR1* could lead to the impaired responsiveness of PSI genes to Pi starvation (Rubio et al., 2001; He et al., 2021). Here, we expressed *PtPHR1* in the *phr1* mutant to generate *PtPHR1OE/phr1* plants, which showed that heterologous expression of *PtPHR1* could partially rescue the phenotype of *phr1* in P-starved conditions (Figure 5). We also observed that the Pi starvation elevated PSI gene expression and anthocyanin content in *PtPHR1OE/phr1* was partially recovered to the level of wild-type plants (Figure 5). These data together indicated that PtPHR1 is a functional homolog of AtPHR1, and this is consistent with the conception that the transcriptional component of the Pi homeostasis regulatory network may be conserve in plants (Zhou et al., 2008). In this study, we demonstrated that PtPHR1 was involved in Pi starvation-induced alkaloid biosynthesis. We found that Pi starvation increased the expression level of *AtAAO4* and the production of BA, whereas these activities were impaired by the loss-function of PHR1 (Figure 6). The findings indicated that Pi starvation-mediated BA biosynthesis was controlled by AtPHR1. Furthermore, suppression of BA biosynthesis in *phr1* mutant could be rescued by the heterologous expression of PtPHR1 (Figure 6), which suggested that PtPHR1 behaved in a manner similar to that of AtPHR1 in the regulation of BA biosynthesis. It has been demonstrated that BA is an intermediate in the formation of alkaloids (Krizevski et al., 2010), and *PtAO4* is also reported to be involved in BA and ephedrine biosynthesis in *P. ternata* (Zhang et al., 2016). Thus, we speculated that PtPHR1 mediated Pi starvation-induced BA-derived alkaloid biosynthesis in *P. ternata*.

Because of its extensive pharmacological activity, increasing concerns have been focused on improving alkaloid production. As the main bioactive compound in *P. ternata*, the research progress in the regulatory of alkaloid metabolism has been described (Miao et al., 2013). Tissue culture materials, such as calluses and protocorm-like bodies, accumulate greater amounts of alkaloid than that of field-grown tubers, and different combinations of 6-benzyladenine, kinetin, α-naphthaleneacetic acid, and 2,4-dichlorophenoxyacetic acid also impaired alkaloid metabolism (Liu et al., 2010, 2015). Application of salicylic acid or methyl jasmonate enhances alkaloids accumulation in *P. ternata* grown in field and suspension tuber, as well as in *in vitro* cultured microtubers (Duan et al., 2017, 2019). In this work, we demonstrated that Pi starvation played a positive role in alkaloid accumulation in *P. ternata*. We also found that Pi starvation-induced BA accumulation in both *P. ternata* and *Arabidopsis* (Figure 6). BA not only severs as intermediate compounds for alkaloid but also is the backbone of numerous compounds in plants, including taxol, cocaine, methylbenzoate, and benzylbenzoate (Ibdah et al., 2009; Del Olmo et al., 2017). BA and its derivatives with high antibacterial and antifungal activity, are commonly used as preservatives and medicines (Del Olmo et al., 2017). Thus, the activation of the BA, as well as alkaloid biosynthesis, by Pi starvation would have a broad ecological and evolutionary consequence on *P. ternata* or closely related species. Also, the regulation of the alkaloid accumulation by the PHRs would provide new insights into the linkage between secondary metabolism and nutrient supply.

## Data availability statement

The data presented in the study are deposited in the GenBank repository, accession number ON075805.

## Author contributions

GH and YH conceived the project, designed the experiments, and wrote the manuscript. HW, JH, and YH carried out the experiments with assistance from LL, XZ, HZ, ZL, and QS. HW, JH, LL, XZ, HZ, ZL, QS, YH, and GH analyzed and discussed the results. All authors contributed to the article and approved the submitted version.

## Funding

This work was funded by the Zhejiang Provincial Natural Science Foundation of China (LR22C020003), the Major Science and Technology Special Project of Variety Breeding of Zhejiang Province (2021C02067-7), National Natural Science Foundation of China (32000234, 31670291, and 31800249), and State Key Laboratory for Managing Biotic and Chemical Threats to the Quality and Safety of Agro-products (2021DG700024-KF202102).

## Conflict of interest

The authors declare that the research was conducted in the absence of any commercial or financial relationships that could be construed as a potential conflict of interest.

## Publisher's note

All claims expressed in this article are solely those of the authors and do not necessarily represent those of their affiliated organizations, or those of the publisher, the editors and the reviewers. Any product that may be evaluated in this article, or claim that may be made by its manufacturer, is not guaranteed or endorsed by the publisher.
