# Corrigendum: Involvement of *PtPHR1* in phosphates starvation-induced alkaloid biosynthesis in *Pinellia ternata* (Thunb.) Breit

**DOI:** 10.3389/fpls.2023.1288386

**Published:** 2023-09-18

**Authors:** Huihui Wang, Jitao Hu, Linying Li, Xueying Zhang, Hao Zhang, Zongsuo Liang, Qing Sheng, Yuqing He, Gaojie Hong

**Affiliations:** ^1^College of Life Sciences, Zhejiang Sci-Tech University, Hangzhou, China; ^2^State Key Laboratory for Managing Biotic and Chemical Threats to the Quality and Safety of Agro-Products, Institute of Virology and Biotechnology, Zhejiang Academy of Agricultural Sciences, Hangzhou, China; ^3^College of Pharmacy, Zhejiang Chinese Medical University, Hangzhou, China

**Keywords:** *Pinellia ternata* (Thunb.) Berit, alkaloid metabolism, benzoic acid (BA), phosphate signaling, *PtPHR1*

In the published article, there was an error in the **Funding** statement. It previously stated “This work was funded by the Zhejiang Provincial Natural Science Foundation of China (LR22C02003).” The correct **Funding** statement appears below:

“This work was funded by the Zhejiang Provincial Natural Science Foundation of China (LR22C020003).”

The authors apologize for this error and state that this does not change the scientific conclusions of the article in any way. The original article has been updated.

