# Supplementary material for: Involvement of PtPHR1 in phosphates starvation-induced alkaloid biosynthesis in Pinellia ternata (Thunb.) Breit
Source: Front Plant Sci. 2022 Aug 10;13:914648. doi: 10.3389/fpls.2022.914648 (PMC9400802; doi:10.3389/fpls.2022.914648)
Supplement: Supplementary file 1 [file Data_Sheet_1.docx]

**Involvement of *PtPHR1* in phosphates starvation-induced** **alkaloid biosynthesis in Pinellia ternata (Thunb.) Breit**

Huihui Wang^#^, Jitao Hu^#^, Linying Li, Xueying Zhang, Hao Zhang, Gaojie Hong.

Authors for correspondence:

Gaojie Hong

Email: gjhong@126.com

Yuqing He

Email: yqhe_123@126.com

**
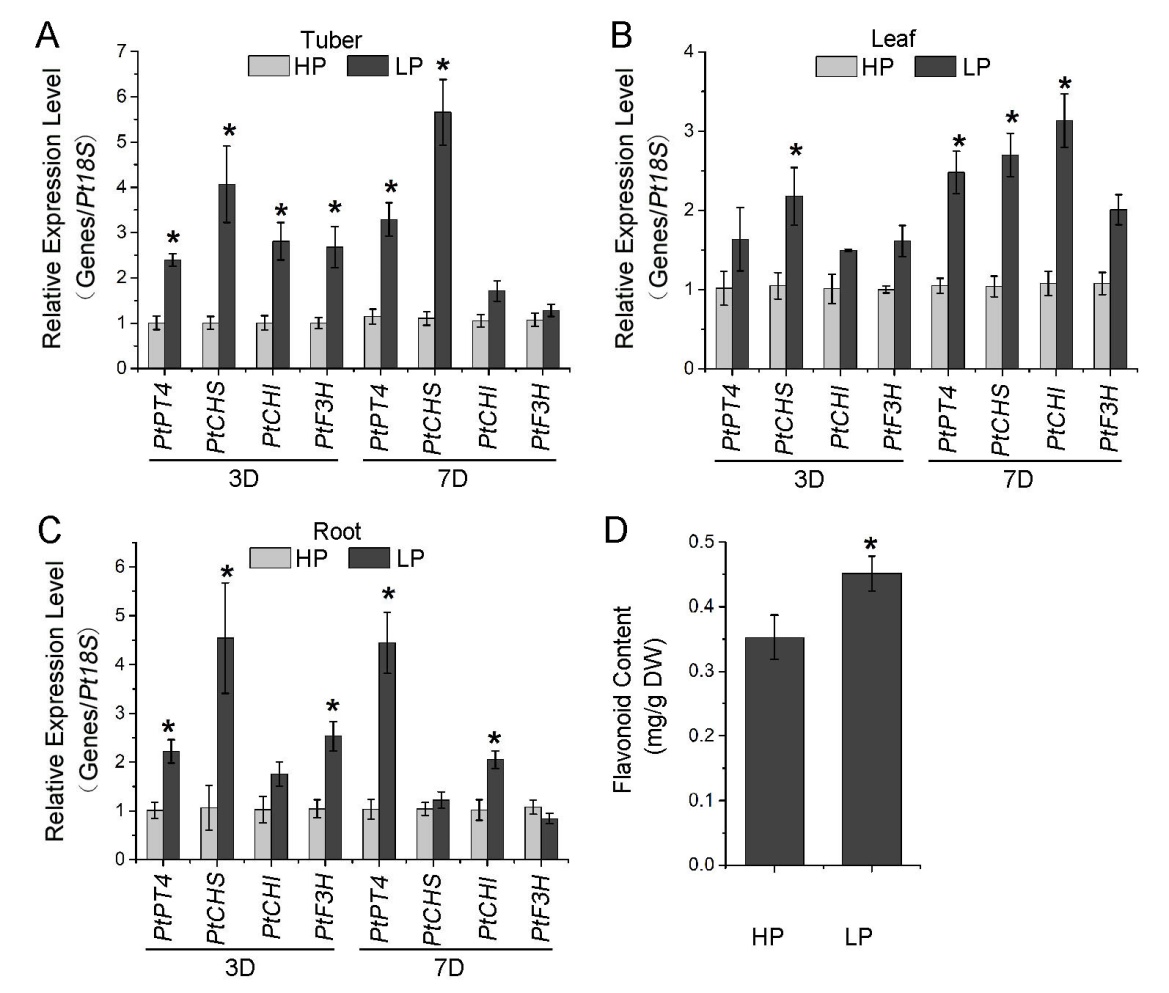
**

**Figure S1 Effect of Pi starvation on flavonoid biosynthesis in *P. ternata*.**

RT-qPCR analysis of alkaloid-responsive genes in *P. ternata* tubers **(A)**, leaves **(B)** and roots **(C)** upon low Pi treatment for 3 and 7 days, respectively. *PtPT4* is a marker gene for Pi starvation. Values are means ± SD of three biological replicates. * indicates significant difference between high/low Pi treatment at *P* < 0.05 by Student’s *t*-test. **(D)** Induction of total flavonoid by Pi starvation treatment in *P. ternata* tuber. Values are means ± SD of three biological replicates. * *P* < 0.05, Student’s *t*-test. DW：dry weight. HP: 10 mM Pi. LP: 10 uM Pi.

**
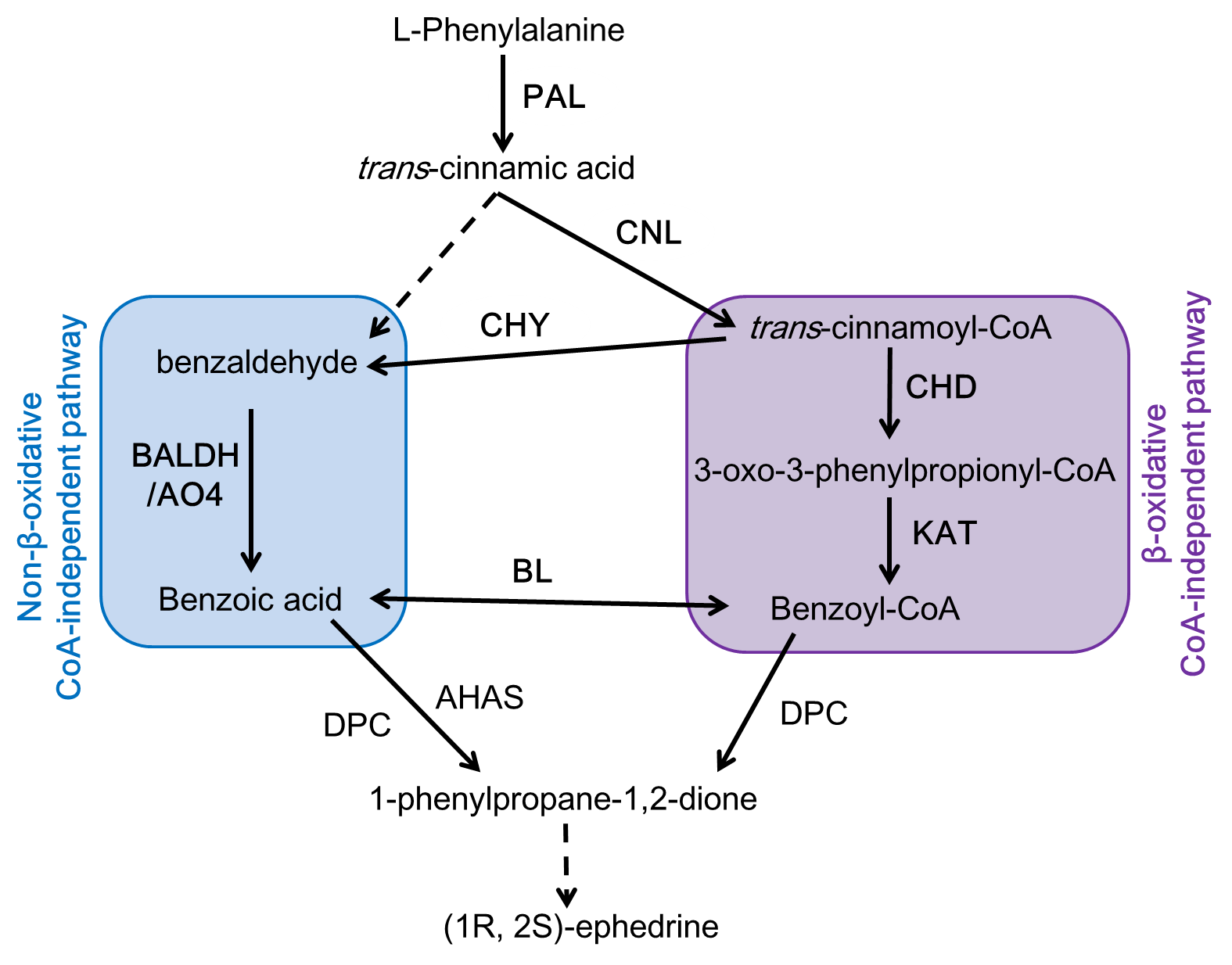
**

**Figure S2.** **Schematic diagram of ephedrine biosynthetic pathway.**

PAL, phenylalanine ammonia lyase; CNL, cinnamate:CoA ligase; CHD, cinnamoyl-CoA hydratase-dehydrogenase; CHY, 3-hydroxyisobutyryl-CoA hydrolase; AO4, aldehyde oxidases 4; KAT, 3-ketoacyl-CoA thiolase; BALDH, benzaldehyde dehydrogenase; BL, benzoate-CoA ligase; DPC, ThDP-dependent pyruvate decarboxylase; AHAS, acetolactate synthase.


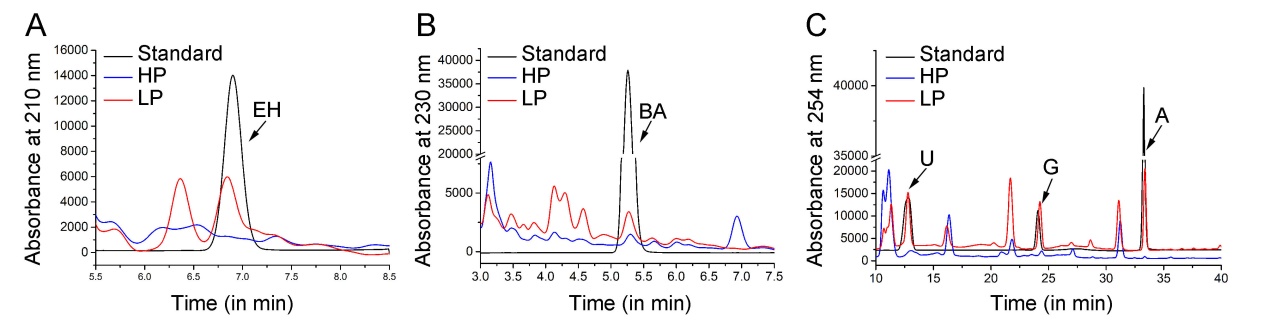


**Figure S3. HPLC analysis of alkaloid compounds extracted from *P. ternata* tubers grown on high and low Pi medium for 7 days.** Relative abundance is shown in arbitrary units, based on absorbance at 210 for ephedrine hydrochloride (EH, y = 17025x + 1996.3, R² = 0.9999), 230 nm for benzoic acid (BA, y = 19777x - 128.9, R² = 0.9984) and 254 nm for guanosine (G, y = 43700x + 12591, R² = 0.9996), adenosine (A, y = 63149x – 18385, R² = 0.9998), and uridine (U, y = 44989x + 376, R² = 0.9999).


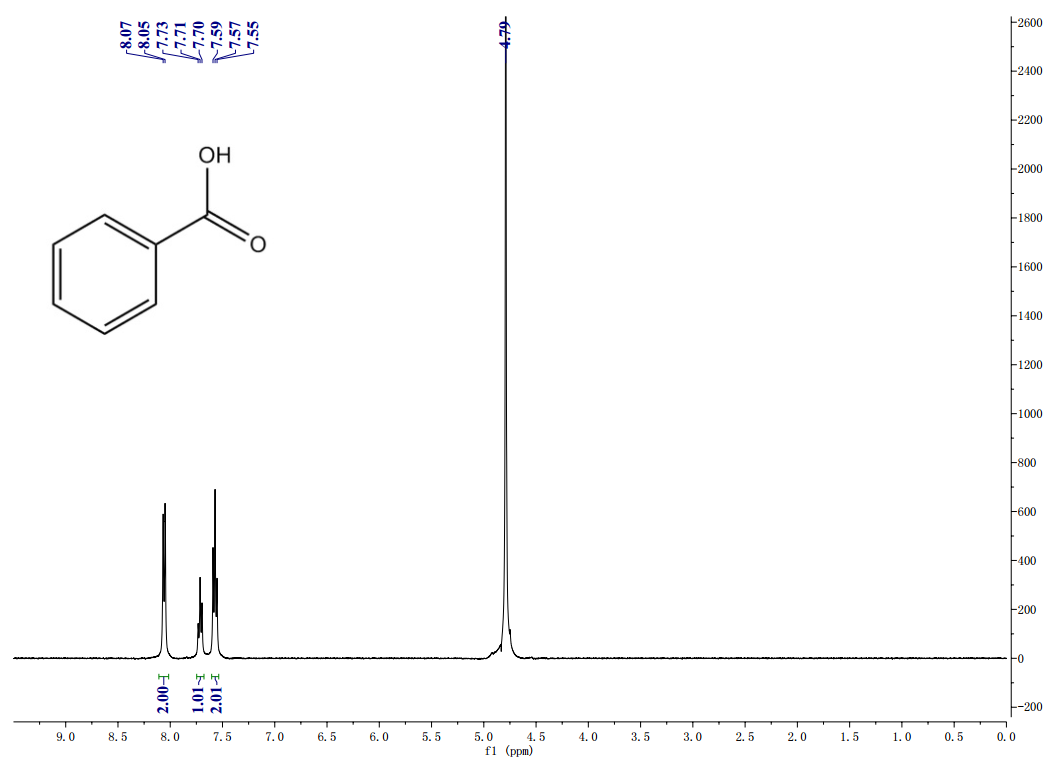


**Figure S4. Validation of benzoic acid standard by Nuclear Magnetic Resonance spectroscopy.**


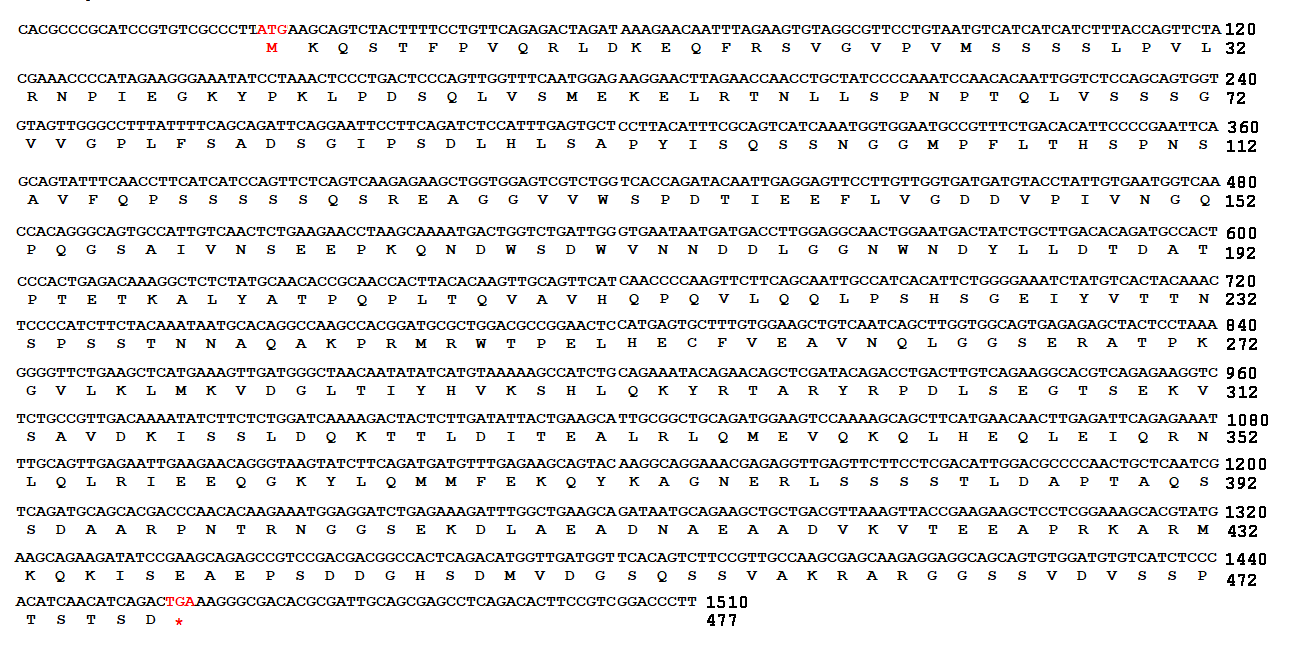


**Figure S5. DNA and amimo acid sequence of the *PtPHR1*.**


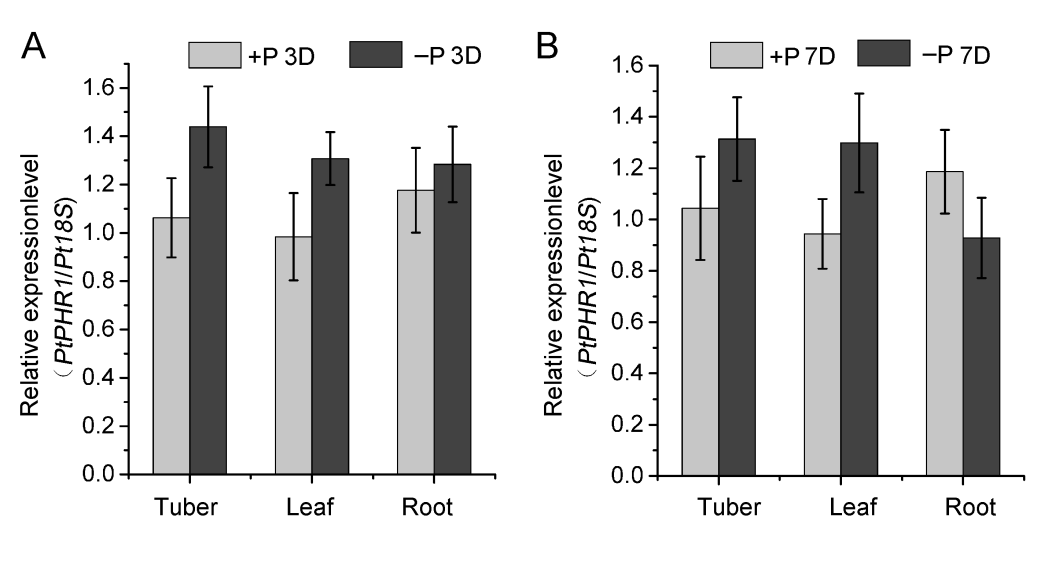


**Figure S6. RT-qPCR analysis of *PtPHR1* transcript level upon Pi starvation.**

Tubers, leaves and roots of *P. ternata* grown in high and low Pi for 3 (A) and 7 (B) days were collected. Values are means ± SD of three biological replicates.


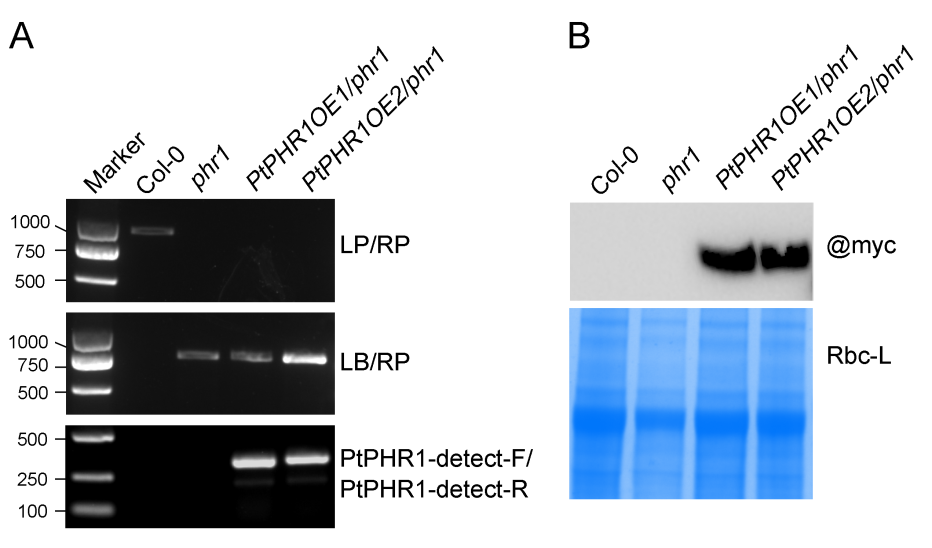


**Figure S7. Identification of *PtPHR1OE/phr1* plants.** **(A)** Genotyping of indicated plants by PCR assay. Upper bands are product of gene-specific primers LP and RP. Middle bands are product of gene-specific primer RP and T-DNA border primer LB. Lower bands are product of gene-specific primer PtPHR1-detected-F and PtPHR1-detected-R. **(B)** Protein levels of PtPHR1 in 9-day-old plants by Western blot assay. The PtPHR1 protein was detected with anti-myc antibody and the Coomassie Brilliant Blue-stained Rubisco large subunit (Rbc L) was used as a loading control.


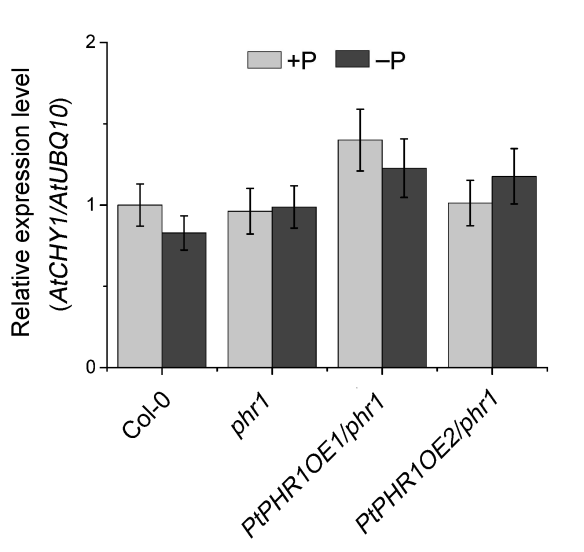


**Figure S8. Expression level of AtCHY1 in 9-day-old Col-0, *phr1* and *PtPHR1OE/phr1* seedlings grown in +P and –P conditions.** The transcript levels were analyzed by qRT-PCR. Values are means ± SD of three biological replicates.


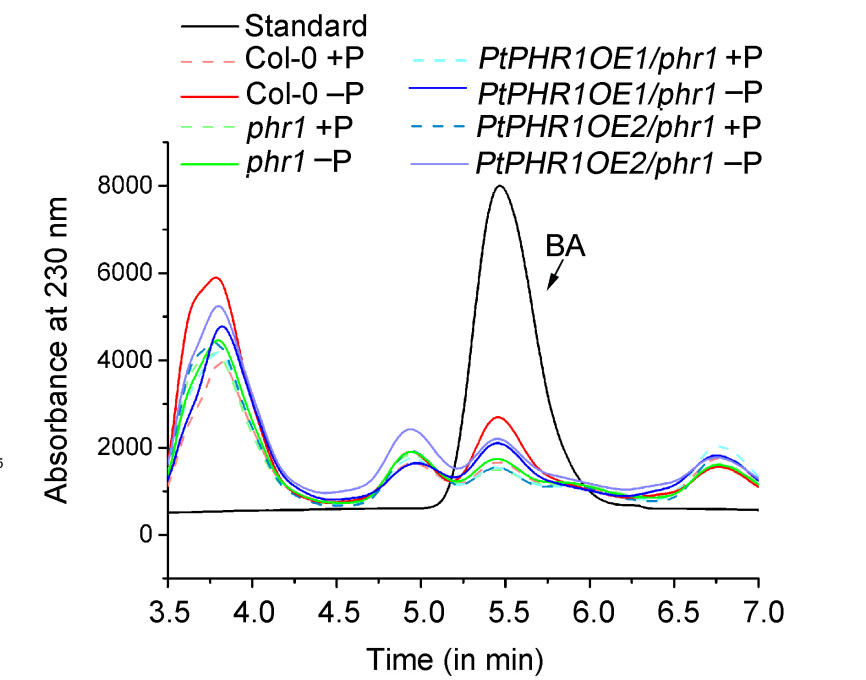


**Figure S9. HPLC analysis of BA extracted from seedlings of Col-0, *phr1* and *PtPHR1OE/phr1* upon Pi starvation treatment*.*** Relative abundance is shown in arbitrary units, based on absorbance at 230 nm BA. y = 108260x - 4968.1, R² = 0.9998.

| **Table S1. Primers used in this work.** | | |
| --- | --- | --- |
| Primer names | Primer sequence F (5'--3') | Primer sequence R (5'--3') |
| RT-PtPAL^1^ | GTCCTGCTTCGGCTTCGTCA | CCAACATCCTGGCTCTGCTCTC |
| RT-PtCNL^1^ | GTGGTGCCAACTTTGTGCCC | GATGGAGGTGCGTTGGGAGT |
| RT-PtCHD^1^ | GTCTCGGTGTAGGCGCTCT | GAGTTCACGGGAGGGTTCA |
| RT-PtKAT^1^ | TGATCCTGTTCTTGGCGTGTA | CTTGGGAGGGGTCAATAAATC |
| RT-PtBALDH^1^ | ACCTGATGGGACTTTTGCTT | TTCCATGAGGACAACTGCTT |
| RT-PtCHY^1^ | CCATCCTATCCTTTCGCTGAC | TGCTGGGGCTTCCTTCTATCT |
| RT-PtAO4^1^ | GGTGCAGCCATAAGTGAAGT | CCATCTGAGTTTGCGAGGTA |
| RT-PtPDC^1^ | AGGGCGGTGTAGTTCCAGTTC | CGGCGAGTTGCGGATATGAGT |
| RT-PtAHAS^1^ | TGTCCTCTTCTCCGACTCCA | GCTCGTTGGGACCGTATCT |
| RT-PtPT1 | ACCACCACCACCTGGTTCCTGT | TGTTCATGGTCGCAGCCTTGGG |
| RT-PtPT4 | CAGAAGCAGCATGGGTGTCGGT | GGGGCAACCCGAAGTCCAAAGA |
| RT-PtCHI^1^ | CGTCGTCGGAGAACGCAATCGT | CCGACGCCGTGGACAAGTTCAA |
| RT-PtF3H^1^ | TTGCCGCCGGACATGTCGAA | TGCCGTAAGATAGTGGCGGCCT |
| RT-PtCHI^1^ | AGACCCCGTTCTCGCCGATGAT | TTGCGTTCTCCGACGACGGTT |
| Pt18S^2^ | CGCATATAAATAAACGGAGGAA | GACGCTTCTACAGACTACA |
| PtCHS^2^ | GCTCAAAGAGTACGGCAACAT | TTCCTTCCTCCTCCGACTTC |
| RT-AtAAO4 | TGGCGCAAAAGGGGGATTTCTCG | TCCCTGCAACCTCAACCGCAAC |
| AtUBQ10 | GGCCTTGTATAATCCCTGATGAAT | GAGATAACAGGAACGGAAACATA |
| RT-AtIPS1 | AGACTGCAGAAGGCTGATTCAGA | TTGCCCAATTTCTAGAGGGAGA |
| RT-AtDFR | AGCCGCCAAGGGACGTTATATTTG | CCGGGAGAAAACCCTTTTGACGA |
| RT-AtAtPAL | CTTGGAACAGAGCTTTTGACCG | CGTGAAAACCTTGTCGAACTCTTC |
| RT-AtCHY1 | TGGGGCAGTGTCTTATCCGCGA | ATGTCCTCCAGTCGCCTTGGCT |
| Rec-AtPHR1 | GAATTCCACCCAAGCAGTGGTATCAACGCAGAGTATGGAGGCTCGTCCAGTTCAT | CCCTCTAGAGGCCGAGGCGGCCGACTTAATTATCGATTTTGGGACGCT |
| Rec-PtPHR1 | GAATTCCACCCAAGCAGTGGTATCAACGCAGAGTATGAAGCAGTCTACTTTTCC | CCCTCTAGAGGCCGAGGCGGCCGACTGTCTGATGTTGATGTGGGAG |
| PtPHR1-detect | TTGCCAAGCGAGCAAGAGGAGG | AAGCGCTACCGTTCAAGTCTTC |
| PtPHR1-myc | CgACgACAAgACCgTCACCATGAAGCAGTCTACTTTTCC | gAggAgAagAgCCgTCgGTCTGATGTTGATGTGGGAG |
| PtPHR1-GFP | GACTCTAGACCCCTGGGATCCATGAAGCAGTCTACTTTTCC | CGCCCTTGCTCACCATGTCGACGTCTGATGTTGATGTGGGAG |
| Hygro | ATGAAAAAGCCTGAACTCACCGCG | TTGCCCTCGGACGAGTGCTG |

1. Zhang G, Jiang N, Song W, Ma C, Yang S, Chen J (2016) De novo sequencing and tsranscriptome analysis of *Pinellia ternata* Identify the candidate genes involved in the biosynthesis of benzoic acid and ephedrine. Frontier in Plant Science 7: 1209

2. Xue T, Zhang H, Zhang Y, Wei S, Chao Q, Zhu Y, Teng J, Zhang A, Sheng W, Duan Y, Xue J (2019) Full-length transcriptome analysis of shade-induced promotion of tuber production in *Pinellia ternata*. BMC Plant Biol 19: 565
